# Multicenter Study of Pin Site Infections and Skin Complications Following Pinning of Pediatric Supracondylar Humerus Fractures

**DOI:** 10.7759/cureus.911

**Published:** 2016-12-03

**Authors:** Kristen Combs, Steven Frick, Gary Kiebzak

**Affiliations:** 1 Medical Student, University of Central Florida College of Medicine, Orlando, FL; 2 Orthopedic Surgery, Nemours Children's Hospital

**Keywords:** pediatric supracondylar humerus fracture, pin site infection

## Abstract

Introduction: Pediatric supracondylar humerus fractures are the most common elbow fractures in pediatric patients. Surgical fixation using pins is the primary treatment for displaced fractures. Pin site infections may follow supracondylar humerus fracture fixation; the previously reported incidence rate in the literature is 2.34%, but there is significant variability in reported incidence rates of pin site infection. This study aims to define the incidence rate and determine pre-, peri-, and postoperative factors that may contribute to pin site infection following operative reduction, pinning, and casting.

Methods: A retrospective chart analysis was performed over a one-year period on patients that developed pin site infection. A cast care form was added to Nemours’ electronic medical records (EMR) system (Epic Systems Corp., Verona, WI) to identify pin site infections for retrospective review. The cast care form noted any inflamed or infected pins. Patients with inflamed or infected pin sites underwent a detailed chart review. Preoperative antibiotic use, number and size of pins used, method of postoperative immobilization, pin dressings, whether postoperative immobilization was changed prior to pin removal, and length of time pins were in place was recorded.

Results: A total of 369 patients underwent operative reduction, pinning, and casting. Three patients developed a pin site infection. The pin site infection incidence rate was 3/369=0.81%. Descriptive statistics were reported for the three patients that developed pin site infections and three patients that developed pin site complications.

Conclusion: Pin site infection development is low. Factors that may contribute to the development of pin site infection include preoperative antibiotic use, length of time pins are left in, and changing the cast prior to pin removal.

## Introduction

Pediatric supracondylar humerus fractures (PSCHF) constitute approximately 17% of all fractures in children and are the most common elbow fracture [1-2]. Type I fractures (nondisplaced) are managed nonsurgically, but most displaced injuries (types III and IV) require surgical intervention [3]. Angulated fracture (type II) treatment is controversial and dependent on the amount of angulation; some advocate for closed treatment and others for surgical treatment. Closed reduction and percutaneous pinning using Kirschner wires (K-wires) is the primary surgical treatment for PSCHF. The most common indications for open reduction are inability to obtain an acceptable closed reduction, entrapment of nerve or artery, and open fractures. Fixation is typically the same with percutaneously placed K-wires [4-5].

Pin site infection after PSCHF may occur when pins are buried underneath the skin or when the ends of the pins are left exposed [6]. Some studies have reported no pin site infection following K-wire fixation, but the average incidence reported is 2.34% (45/1,922) [7]. The range in reported incidence rates may be attributable to variable pre-, peri-, and postoperative factors.

Postoperatively, casts or splints are typically used, but studies have not shown an association between a specific type of immobilization associated with a higher incidence of pin site infections [8]. In addition to the type of cast, cast care may affect the development of pin site infection. Patient education and compliance may be a factor, as one study reports 60% of patients who developed pin site infections had a history of missed appointments or wet dressings [9].

The type of skin preparation and draping used for closed reduction and pinning, and the dressing used to cover the pin site after surgery, vary in use after PSCHF. Percutaneous pinning using a semi-sterile surgical technique reported no incidence of pin site infection, with historical controls being sterile surgical preparation and draping. The semi-sterile technique saves time and money compared to a sterile surgical technique, which is currently commonplace [7]. The study found that the administration of perioperative antibiotics may not be necessary as 68% of the patients did not receive any antibiotics during the preoperative or postoperative period, and no pin site infections developed.

A significant risk factor for pin infection is the length of time the pins are left in place, as longer periods of time increase the opportunity time for an infection to develop [6]. Typically PSCHFs unite rapidly and pins can be removed at three or four weeks after surgery. In other areas of the body (distal femur) and other elbow fractures (lateral condyle fractures), it is generally accepted that K-wires not buried beneath the skin should be removed by six weeks to lessen the chance of infection [6].

A gap in the literature exists due to the variability in reported incidence rates of pin site infection following percutaneous pinning to treat PSCHFs. This study seeks to analyze data collected at the time of pin and cast removal following surgical treatment of PSCHF to define the incidence rate of pin site infection and skin complications following percutaneous pin fixation. A retrospective chart review of identified cases was conducted to evaluate the pre-, peri-, and postoperative factors that contribute to the development of pin site infections and complications. The null hypothesis states that there is no difference in the reported incidence rate of pin site infection in the literature and the study incidence rate. The alternative hypothesis states that the study incidence rate of pin site infection is lower than the pin site infection rate reported in the literature.

## Materials and methods

Approval for this study was obtained from Nemours Institutional Review Board (approval #720767). Patient informed consent was obtained.

A retrospective chart analysis was performed on pediatric patients (age range: one to 18 years) who were identified with pin site infection and complication following supracondylar humerus fracture fixation. Patients who had supracondylar humerus fracture fixation that was performed at Nemours’ clinical sites in Orlando, FL, Jacksonville, FL, Pensacola, FL, or Wilmington, DE, and developed pin site infection, were included in this study. There were no specific exclusion criteria other than age. Data collection for this study ranged from December 1, 2014, through December 1, 2015.

Patient charts were accessed using Nemours' EMR system. A standardized cast form within the medical record was used to identify patients who developed pin site infection upon cast removal. This form was completed by an orthopedic technician at the time of cast removal on every patient in the system, and it specifically collected data on the presence or absence of pins beneath a cast. For each patient with K-wires beneath the cast, the form requires evaluation of the pin site either as normal, inflamed, or infected. Inflamed pins are those that are erythematous or have granulation tissue present, but do not require antibiotic treatment and are treated only with pin removal. Any pin site that is treated with oral or intravenous antibiotics or with surgical debridement, is defined as infected. To establish a denominator and calculate the incidence of pin site infection, all patients with PSCHFs who underwent surgery at the four sites were identified by a search of our coding system for the current procedural terminology (CPT) codes for closed and open treatment of supracondylar humerus fractures (24538, 24545, and 24546). Wound healing complications were recorded if occurring in the 30 days following pin removal. Data were abstracted into an Excel spreadsheet. Data collected for analysis were diagnosis, type of fracture (open or closed, and grade: Gartland type II, III, or IV), age, sex, race, type of surgical preparation used, draping types, antibiotic used, and timing. Details about pin use were recorded: pin size, number of pins, pin entry site (lateral or medial), pin site dressing, and pin site care instructions given to the patient [10]. Perioperative antibiotic use (dose, type, and administration timing) was recorded, as well as type of cast or splint (and whether changed prior to pin removal), and number of days the pins were left in. Patients were de-identified using unique study numbers. Data were recorded on a password-protected Excel spreadsheet.

Descriptive statistics were calculated. The incidence rate of pin site infection was expressed as a frequency and percentage. Our pin site infection rate was compared to historic data from Iobst et al. [7], who performed a literature search and summarized results from 22 studies that reported the incidence of infection after percutaneous pinning of PSCHFs, with an overall infection rate of 2.34% (45 pin site infections in 1,922 reported cases). A chi-square analysis was performed with an alpha level equal to 0.05 to demonstrate statistical significance. A post hoc power analysis was performed to estimate the power of the comparison using StatMate (GraphPad Software, La Jolla, CA).

## Results

Three hundred sixty-nine pediatric patients underwent surgical reduction and pin fixation for PSCHF during the data collection period. Three patients developed a pin site infection for a 0.81% (3/369) incidence rate. Our rate of 0.81% was not statistically significantly different from the rate of 2.34% reported by Iobst et al. (chi-square, P=0.093, alpha=0.05). A post hoc power analysis suggested the power to detect the difference in infection rates (1.53%) was about 55%.

A detailed chart review of the three patients who developed a pin site infection was performed. Additionally, three patients whose pin site was noted to be inflamed developed an alteration in skin integrity (pressure sore or ulcer). Their pin sites were not infected and therefore did not contribute to the reported incidence of pin site infection. A detailed chart review of these inflamed ulcer and pressure sore cases was also performed to determine factors that contribute to altered pin site skin integrity and complication development. Demographic variables of these cases are shown in Tables 1-2. Notable pre-, peri-, and postoperative variables for the presented cases are shown in Tables 3-4. 

**Table 1:** Demographic Variables for Infection Cases

Variable	Case One - Infection	Case Two - Infection	Case Three - Infection
Age (years)	8	7	7
Sex	Male	Male	Male
Race	Non-Hispanic/Latino	Non-Hispanic/Latino	Non-Hispanic/Latino
Type of Fracture	Closed	Closed	Closed

**Table 2:** Demographic Variables for Pressure Sore and Ulcer Cases

Variable	Case Four - Pressure Sore	Case Five - Ulcer	Case Six - Ulcer
Age (years)	9	6	5
Sex	Male	Male	Male
Race	Hispanic/Latino	Hispanic/Latino	Non-Hispanic/Latino
Type of Fracture	Closed	Closed	Closed

**Table 3:** Pre-, Peri-, and Postoperative Variables for Infection Cases

Variable	Case One - Infection	Case Two - Infection	Case Three - Infection
Preoperative Antibiotic Use	No	No	No
Number of Pins	2	2	3
Pin Size	2 mm	1.4 mm	2.0 mm
Pin Site Dressing	Reston Foam and sterile cast padding	Adaptic (4x4) and foam in the antecubital fossa and sterile Webril	Adaptic (4x4) foam and sterile Webril
Cast/Splint Changed Prior to Pin Removal	No	No	Yes
Number of Days Pins Left In	24	28	31
Cast Care Instructions Followed	Yes	Yes	Yes

**Table 4:** Pre-, Peri-, and Postoperative Variables for Pressure Sore and Ulcer Cases

Variable	Case Four - Pressure Sore	Case Five - Ulcer	Case Six - Ulcer
Preoperative Antibiotic Use	No	No	No
Number of Pins	3	2	2
Pin Size	1.6 mm	1.6 mm	2 mm
Pin Site Dressing	Xeroform, sterile dry felt, and Jurgan Balls	4x4 Webril sterile dressing	Adaptic dressing and foam around pins
Cast/Splint Changed Prior to Pin Removal	Yes	No	No
Number of Days Pins Left In	70	32	26
Cast Care Instructions Followed	Yes	No	No

## Discussion

We found the incidence of pin site infections after surgical treatment of PSCHF to be low (3/369=0.81%), and not significantly different from rates reported by others [7]. The power of the comparison was low, however, highlighting that the incidence of pin site infections and skin integrity problems following pinning of PSCHF is so low that an extremely large trial would be needed to demonstrate a high level of statistical power.

There is varied opinion in the literature on the use of preoperative antibiotics in PSCHF surgical procedures. Bashyal et al. found no advantage to the use of preoperative antibiotic use in preventing pin site infections [11]. The three patients who developed pin site infections and the three patients who developed skin integrity complications were not administered preoperative antibiotics.

One patient developed a pressure sore around the pins after presenting late for cast removal (37 days postoperatively). The patient complained that his distal humerus was still tender to palpation when the cast was taken off at this visit. Another cast was applied, and the patient was instructed to come back in one week for final cast and pin removal. The patient was then lost to follow-up for one month, and when the patient presented at clinic, the pins had been in place for 70 days. The suggested time for pin removal following PSCHF is between three and four weeks [12]. This case demonstrates the importance of removing a patient’s pins and cast as soon as medically indicated. The longer the pins are left in, the greater chance of complication development. The family was Spanish speaking and a healthcare provider noted in the patient’s chart that there may have been a misunderstanding in the instructions for follow-up because of the language barrier between the healthcare provider and the family.

Two patients developed superficial ulcers when they presented postoperatively for cast removal. After removing the casts, dirt and water was noted underneath of the casts. Wet casts and foreign objects have both been shown to contribute to the development of infection, skin breakdown, and skin irritation [13]. Furthermore, in Case Four the cotton had been picked out from the inside of the cast, and the father had tried unsuccessfully to remove the cast with scissors and a knife. Both of these cases stress the importance of cast care education following PSCHF. Cast care instructions are given to each patient when he or she is placed into a cast. These instructions make it clear that the cast needs to remain dry, clean, and clear of any foreign objects.

Limitations of this study include the relatively low sample size and resultant low post hoc power. Causation cannot be proven between the previously mentioned variables (pre-operative antibiotic use, duration of time pins left in, and appropriate cast care) and the development of pin site infection and complication. This study only included data from the four Nemours clinical sites in Orlando, FL, Jacksonville, FL, Pensacola, FL, and Wilmington, DE. This data may be representative of the pediatric patients in these locations, but may not be representative of the national pediatric population.

This study reports a very low incidence of pin site infections. The three pin site infection patients did not receive pre-operative antibiotics, and although the use of pre-operative antibiotics may have a role in the prevention of pin site infections following PSCHF, a much larger clinical trial would be needed to verify that assumption. Limiting the length of time that pins are left in is important in the development of pin site complications, and this study provides further evidence to support this practice. Finally, the importance of proper cast-care and patient education is important in the prevention of pin site complications.

## Conclusions

Pin site infection development following PSCHF at Nemours’ clinical sites is low. Factors that may contribute to the development of pin site infection include preoperative antibiotic use, length of time pins are left in, inappropriate cast care, and changing the cast prior to pin removal.
